# The Review of Anti-aging Mechanism of Polyphenols on *Caenorhabditis elegans*

**DOI:** 10.3389/fbioe.2021.635768

**Published:** 2021-07-01

**Authors:** Limin Liu, Peisen Guo, Peixi Wang, Shanqing Zheng, Zhi Qu, Nan Liu

**Affiliations:** ^1^College of Public Health, Zhengzhou University, Zhengzhou, China; ^2^Institute of Chronic Disease Risks Assessment, School of Nursing and Health, Henan University, Kaifeng, China; ^3^School of Basic Medical Sciences, Henan University, Kaifeng, China; ^4^Institute of Environment and Health, South China Hospital, Health Science Center, Shenzhen University, Shenzhen, China

**Keywords:** polyphenols, *Caenorhabditis elegans*, anti-aging, anti-oxidant, insulin/insulin-like signaling pathway

## Abstract

Micronutrients extracted from natural plants or made by biological synthesis are widely used in anti-aging research and applications. Among more than 30 effective anti-aging substances, employing polyphenol organic compounds for modification or delaying of the aging process attracts great interest because of their distinct contribution in the prevention of degenerative diseases, such as cardiovascular disease and cancer. There is a profound potential for polyphenol extracts in the research of aging and the related diseases of the elderly. Previous studies have mainly focused on the properties of polyphenols implicated in free radical scavenging; however, the anti-oxidant effect cannot fully elaborate its biological functions, such as neuroprotection, Aβ protein production, ion channel coupling, and signal transduction pathways. *Caenorhabditis elegans* (*C. elegans*) has been considered as an ideal model organism for exploring the mechanism of anti-aging research and is broadly utilized in screening for natural bioactive substances. In this review, we have described the molecular mechanisms and pathways responsible for the slowdown of aging processes exerted by polyphenols. We also have discussed the possible mechanisms for their anti-oxidant and anti-aging properties in *C. elegans* from the perspective of different classifications of the specific polyphenols, such as flavonols, anthocyanins, flavan-3-ols, hydroxybenzoic acid, hydroxycinnamic acid, and stilbenes.

## Introduction

Aging is considered a universal physiological process that is accompanied by systemic changes in the structural integrity of cells that are caused by alterations in metabolic and signal transduction pathways (Childs et al., 2015). Understanding of the biological mechanisms of aging and longevity has been growing remarkably over the past two decades. At the molecular level, senescence is strongly associated with susceptibility to chronic diseases and disorders, such as chronic fibrosis, severe atherosclerosis, diabetes, osteoarthritis, and ultimately death (Childs et al., 2016; Amor et al., 2020). Among the various anti-aging methods and preventive strategies, the use of micronutrients or biologically active substances is considered a practical and efficient method that targets a variety of intracellular/extracellular pathways (Sahin et al., 2011; Johnson et al., 2013; Li et al., 2017).

Nutrients and bioactive substances have shed new light on the prevention and treatment of chronic diseases and aging. For example, short-term supplementation with appropriate doses of vitamin C or vitamin C plus E has already been confirmed to improve the immunological function in the elderly and contribute to health and longevity (De la Fuente et al., 2020). Most of the substances that exert bioactive properties originate from natural plants and animals and have been extensively studied for their preventive and therapeutic effects against chronic diseases and aging. Functional nutrition is of great significance to human health; however, the high cost involved in extracting and purifying bioactive compounds from natural sources in the past has limited the rapid growth of the market. Thus, with the development of synthetic biology technology, a few important functional nutrients can be produced at a low cost by biological manufacturing. In the future, biological manufacturing is expected to be replaced by traditional extraction techniques or functional nutritional chemicals. So far, plant polyphenols, such as blueberry polyphenols, black tea and green tea polyphenols, and tocotrienols in vegetable oils, have been proved to delay the aging process in model organisms (Adachi and Ishii, 2000; Wilson et al., 2006; Peng et al., 2009; Salminen et al., 2012; Zarse et al., 2012). The anti-aging effects of these polyphenols are mostly related to their anti-oxidant properties and their ability to scavenge free radicals. It has been reported that resveratrol, a polyphenol compound in red wine, could slow down aging in *Caenorhabditis elegans* due to the reduction of mitochondrial respiration (Wood et al., 2004). The understanding of human aging and longevity might be improved by elucidating the molecular mechanism of aging in *C. elegans* (Park et al., 2020).

## Advantages of Using *C. elegans* As a Model Organism in Applied Anti-Aging Research

Although experiment with a mammalian model is compelling, it is time-consuming and limited by the presence of ethical concerns. *C. elegans* has been proved as a reasonable model organism for biological research on aging because of its advantageous features (Guarente and Kenyon, 2000). Although its anatomical structure is simple, the tissues and organs, such as muscles, nervous system, gastrointestinal tract, and gonads of *C. elegans*, are similar to that of higher animals (Jorgensen and Mango, 2002). In addition, its complete genome sequence is available, and about 50% of human protein-coding sequences have identifiable homologous genes in nematodes (Kim et al., 2018). Similar to humans and other higher mammals, its behavior changes and descending physiological indexes are accompanied by aging. Moreover, there are highly evolutionary conserved mechanisms controlling physiological phenomena, such as development, aging, and disease. Homologous or functionally similar forms of the main enzymes, genes, and transcription factors involved in metabolism have been found in higher animals and *C. elegans* (Chen et al., 2013). For example, the important transcription factor forkhead box O (FOXO), which is involved in longevity, stress resistance, and metabolism, is present in drosophila, nematodes, rodents, and humans (Martins et al., 2016). Therefore, *C. elegans* is broadly utilized in screening for natural bioactive substances (Ye et al., 2020). Numerous transgenes and mutants related to the longevity and aging of *C. elegans* are available (Chen et al., 2015), and many polyphenols have been successfully tested for their effects on general health benefits and longevity on nematodes.

At present, most of the bioactive substances with anti-aging activity were first discovered by using nematodes as model organisms. Since the first use of nematodes by Brenner as a tool in genetics research (Brenner, 1974), the model has been applied to many other research fields, such as development, disease modeling, metabolism, medicine, screening, and others. We also took advantages of this model organism in aging and signal transduction (Zheng et al., 2018; Qu et al., 2020b). Since two American scientists, Friedman and Johnson, discovered in the 1980s that the mutation of a single gene in nematodes can increase lifespan (Johnson and Wood, 1982; Friedman and Johnson, 1988), the genetic control of aging has rapidly developed. It is reported that aging and aging-related diseases are controlled by signaling pathways, such as autophagy-related target of rapamycin (TOR) signaling pathway (McCormick et al., 2011; Laplante and Sabatini, 2012), insulin/insulin-like growth factor 1 (IGF-1) signaling (IIS) pathway (Barbieri et al., 2003; Lapierre and Hansen, 2012), mitochondrial-related functional signaling pathway (Sohal and Orr, 2012), and adenosine monophosphate (AMP)-activated protein kinase (AMPK) signaling pathway related to cell energy homeostasis (Salminen and Kaarniranta, 2012; Qu et al., 2020a).

## Polyphenols

**Figure 1:**
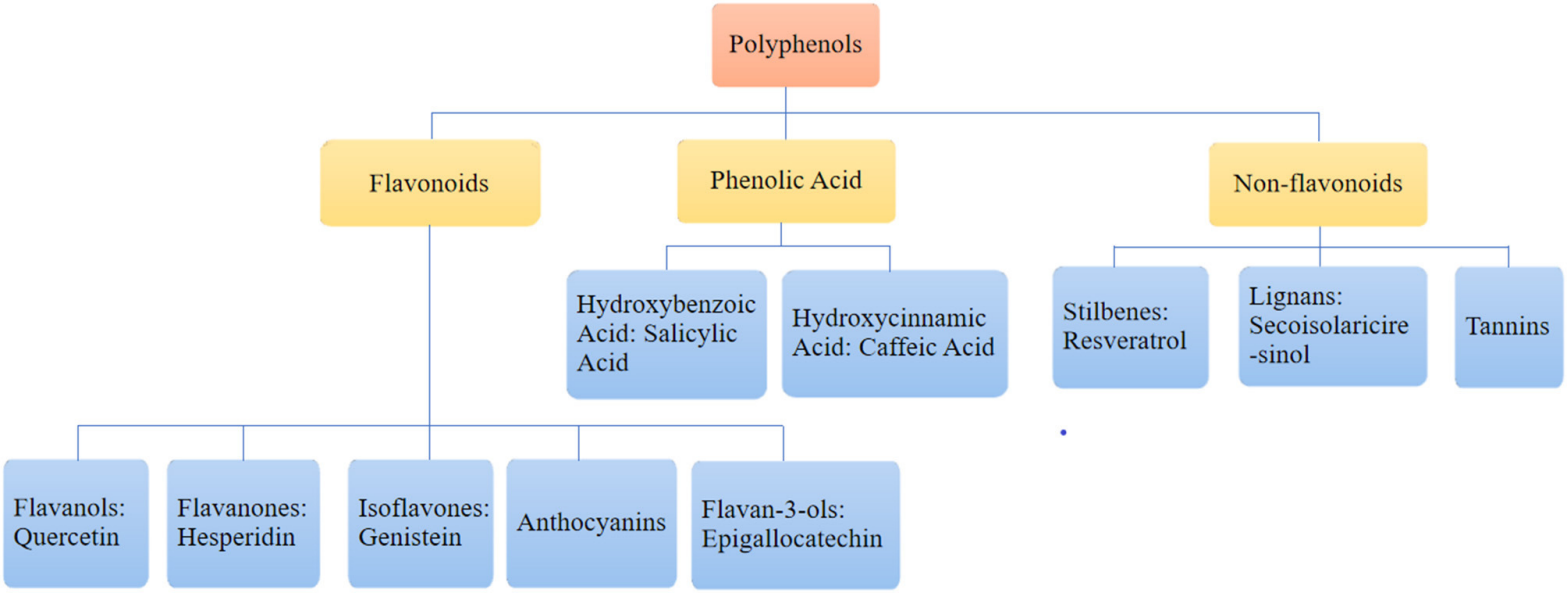
The category of polyphenols.

Polyphenols are the most widely distributed group of phytochemicals (Table 1). They are usually classified into flavonoids, phenolic acids, and non-flavonoids. Flavonoids are subdivided into flavonols, flavanones, isoflavones, anthocyanins, and flavan-3-ols according to their chemical structure. Phenolic acids are subdivided into hydroxybenzoic acid and hydroxycinnamic acid. Non-flavonoids are subdivided into lignans, stilbenes, and tannins (Papaevgeniou and Chondrogianni, 2018; Fraga et al., 2019; Majidinia et al., 2019). The category is illustrated in Figure 1.

**Table 1:** Modulation of the lifespan in *Caenorhabditis elegans* by polyphenols.

**Compound**	**Chemical Structure**	**Source**	**Pathway/regulator**
**Flavonols**			
Quercetin	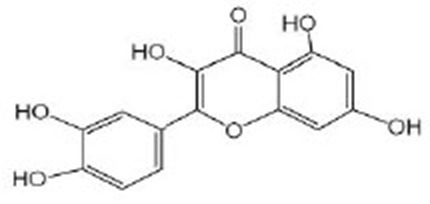	Quercetin was purchased from Sigma-Aldrich (Deisenhofen, Germany) (Kampkotter et al., 2008; Pietsch et al., 2009) Quercetin was purchased from Cayman Chemical Company (Ann Arbor, MI, USA) (Sugawara and Sakamoto, 2020)	IIS/DAF-2/AGE-1, MAPK/UNC-43/SEK-1, DAF-16, HSF-1, SKN-1
Myricetin	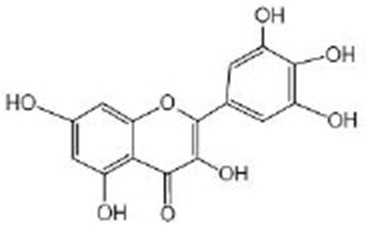	Myricetin was purchased from Sigma-Aldrich (Deisenhofen, Germany) (Grunz et al., 2012). Myricetin was obtained from Extrasynthese, Genay, France (Buchter et al., 2013) LC–MS/MS^A^ proved that the ethyl acetate fraction of *Eugenia uniflora* is rich in myricetin (Sobeh et al., 2020)	DAF-16
Baicalein	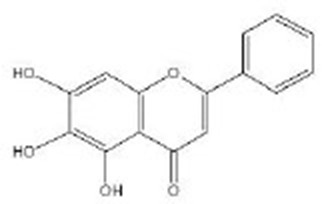	Baicalein (≥98%) was obtained from Sigma-Aldrich (Deisenhofen, Germany) (Havermann et al., 2013, 2016)	SKN-1
Anthocyanins	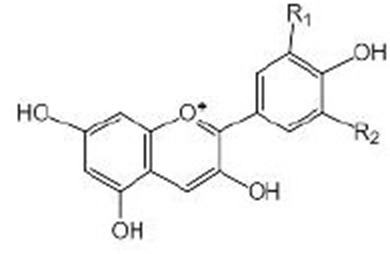	Anthocyanin contents of the purple wheat and acai extracts were characterized and verified by HPLC–UV/vis^B^ (Chen et al., 2013; Peixoto et al., 2016). Anthocyanins extract from bilberry fruit were analyzed by HPLC-DAD^C^ and ESI-MS^D^ (Gonzalez-Paramas et al., 2020). The purple pitanga extract is rich in anthocyanin, mainly in cyanindin-3-O-glucoside (Tambara et al., 2018) Previous HPLC-MS^E^ results proved that the extract of tart cherry (TCE) was rich in anthocyanins, and the concentration of TCE was determined according to the concentration of anthocyanins in a previous experiment (Jayarathne et al., 2020) Mulberry anthocyanins were conducted by HPLC^F^ (Yan et al., 2017)	DAF-16, AAK-2, SKN-1, PMK-1
**Flavan-3-ols**			
Catechin acid	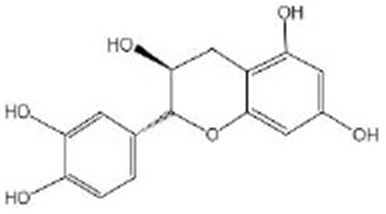	Catechin acid (96%, HPLC) was obtained from Sigma-Aldrich (St Louis, MO, USA) (Wu et al., 2020)	BEC-1, PINK-1
Epigallocatechin-3-gallate (EGCG)	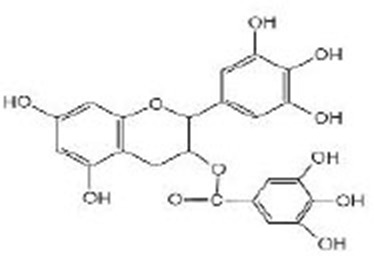	EGCG were obtained from Sigma-Aldrich (St. Louis, MO, USA) (Brown et al., 2006; Zhang et al., 2009; Bartholome et al., 2010; Xiong et al., 2018)	AAK-2, SIR-2.1, DAF-16
**Hydroxybenzoic acid**			
4-Hydroxybenzoic acid	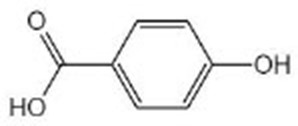	4-Hydroxybenzoic acid with purity >98% from extract of *Veronica peregrina* was verified by HPLC (Kim et al., 2014)	SIR-2.1, DAF-16
Aspirin	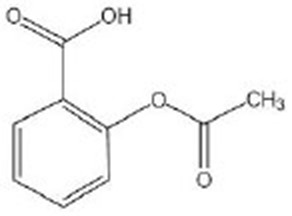	Aspirin was purchased from Sigma-Aldrich (St. Louis, MO, USA) (Huang et al., 2017)	DAF-12, DAF-16
**Hydroxycinnamic acid**			
Chlorogenic acids (CGA)	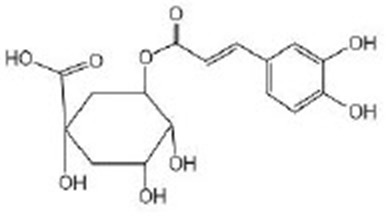	NMR^G^ and UPLC/ESI-HRMS^H^ analyses of green coffee extract confirmed a significant content of CGA (Amigoni et al., 2017) CGA was purchased from Adamas (Basel, Switzerland) (Zheng et al., 2017)	IIS/AKT/DAF-16
5-O-caffeoylquinic acid (5-CQA)	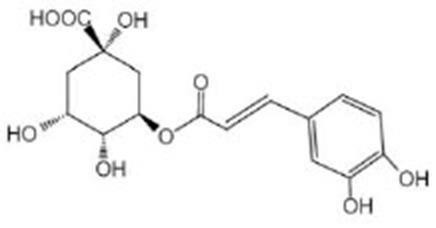	5-CQA is the isomer with the highest content of chlorogenic acid in green coffee extract (Amigoni et al., 2017) 5-CQA were purchased from Aladdin (Shanghai, China) (Zheng et al., 2017)	IIS/AKT/DAF-16
*p*-Coumaric acid	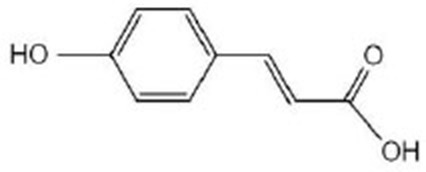	*p*-Coumaric acid (≥98% pure) was purchased from Sigma-Aldrich (St. Louis, MO, USA) (Yue et al., 2019)	SKN-1, OSR-1
**Lignans**			
Sesamin	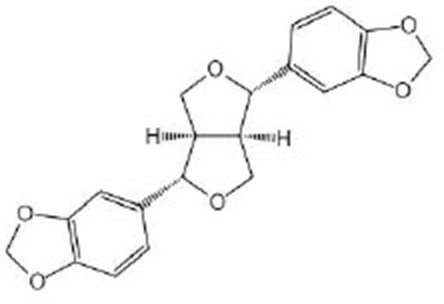	Sesamin was purchased from Wako (Osaka, Japan). γ-Cyclodextrin (γCD) was obtained from Cyclochem (Kobe, Japan) (Yaguchi et al., 2014; Nakatani et al., 2018)	IIS/DAF-2/DAF-16, MAPK/PMK-1/SKN-1, TOR/DAF-15, SIR-2.1, AAK-2, BEC-1
**Stilbenes**			
Resveratrol	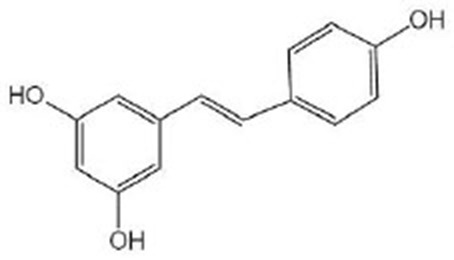	Resveratrol was from purchased from Sigma-Aldrich (St. Louis, MO, USA) (Morselli et al., 2010; Lee et al., 2016; Yoon et al., 2019)	SIR-2.1, DAF-16, AAK-2, MPK-1, BEC-1
**Tannins**			
Tannic acid	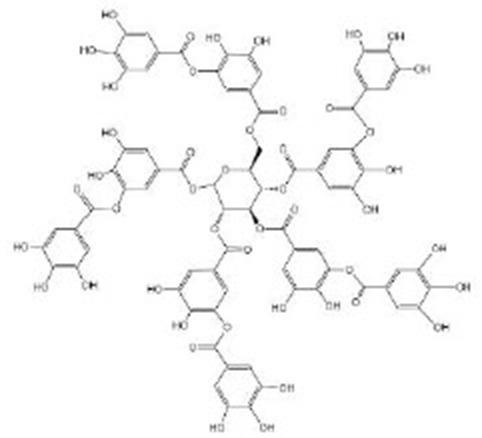	Tannic acid was purchased from Sigma-Aldrich (Taufkirchen, Germany) (Saul et al., 2010, 2011)	SEK-1, *eat-2*
Oenothein B (OEB)	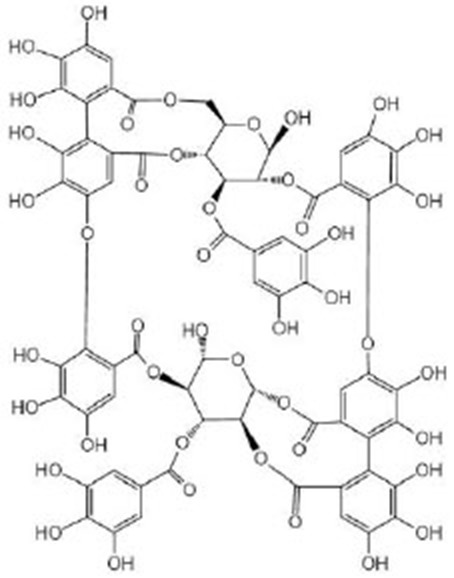	OEB was isolated and purified from eucalyptus leaves, and the isolated and purified OEB was confirmed by HPLC and ^1^H NMR^I^ (Chen et al., 2020)	IIS/AGE-1/DAF-16, SIR-2.1, *eat-2, isp-1*
Pentagalloyl glucose (PGG)	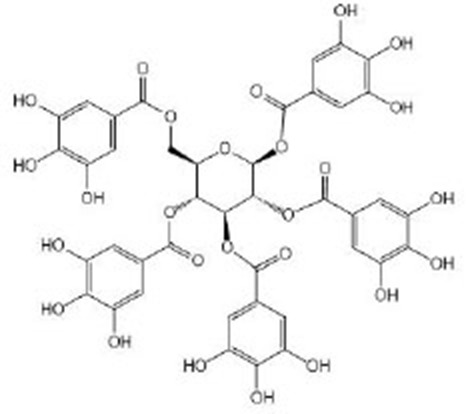	PGG was isolated and purified from eucalyptus leaves and was confirmed by HPLC and MS^J^ (Chen et al., 2014)	IIS/AGE-1/DAF-16, SIR-2.1, *eat-2, isp-1*

*A: LC-MS/MS, liquid chromatograph-mass spectrometer; B: HPLC–UV/vis, high-performance liquid chromatography–ultraviolet/visible spectroscopy; C: HPLC-DAD, high-performance liquid chromatography-diode array detector; D: ESI-MS, electrospray ionization mass spectrometry; E: HPLC-MS, high-performance liquid chromatography-mass spectrometry; F: HPLC, high-performance liquid chromatography; G: NMR, nuclear magnetic resonance spectroscopy; H: UPLC/ESI-HRMS, hybrid quadrupole-orbitrap mass spectrometry; I: ^1^H NMR, ^1^H nuclear magnetic resonance spectroscopy; J: MS, mass spectrometer*.

Polyphenols exert beneficial effects on health, owing to their anti-oxidant and anti-inflammatory activities, and they have been commonly employed to treat cancer, autoimmune diseases, type 2 diabetes, cardiovascular disorders, and other diseases. The structural characteristics of the carbocyclic ring of polyphenols and the number of hydroxyl groups on the ring are the main prerequisites for prolonging lifespan (Grunz et al., 2012). In this article, we reviewed literature regarding the anti-aging properties of each specific polyphenol. Different classes of chemicals might activate similar signaling pathways involved in aging processes, and one class of chemicals might be involved in multiple pathways. For example, it was reported that resveratrol can extend *C. elegans* lifespan through the MPK-1/ERK or SIR-2.1/DAF-16 pathway (Yoon et al., 2019). In addition, many kinds of polyphenols can modulate longevity through the IIS pathway, especially through the key transcription factor DAF-16 in the pathway, for example, myricetin (Buchter et al., 2013), blueberry extract (Wang et al., 2018), echinacoside (Wang et al., 2015), and others. The main reason for this effect might be that the DAF-16 expression increases the ability to scavenge free radicals and resist oxidative stress.

## Flavonols

**Figure 2:**
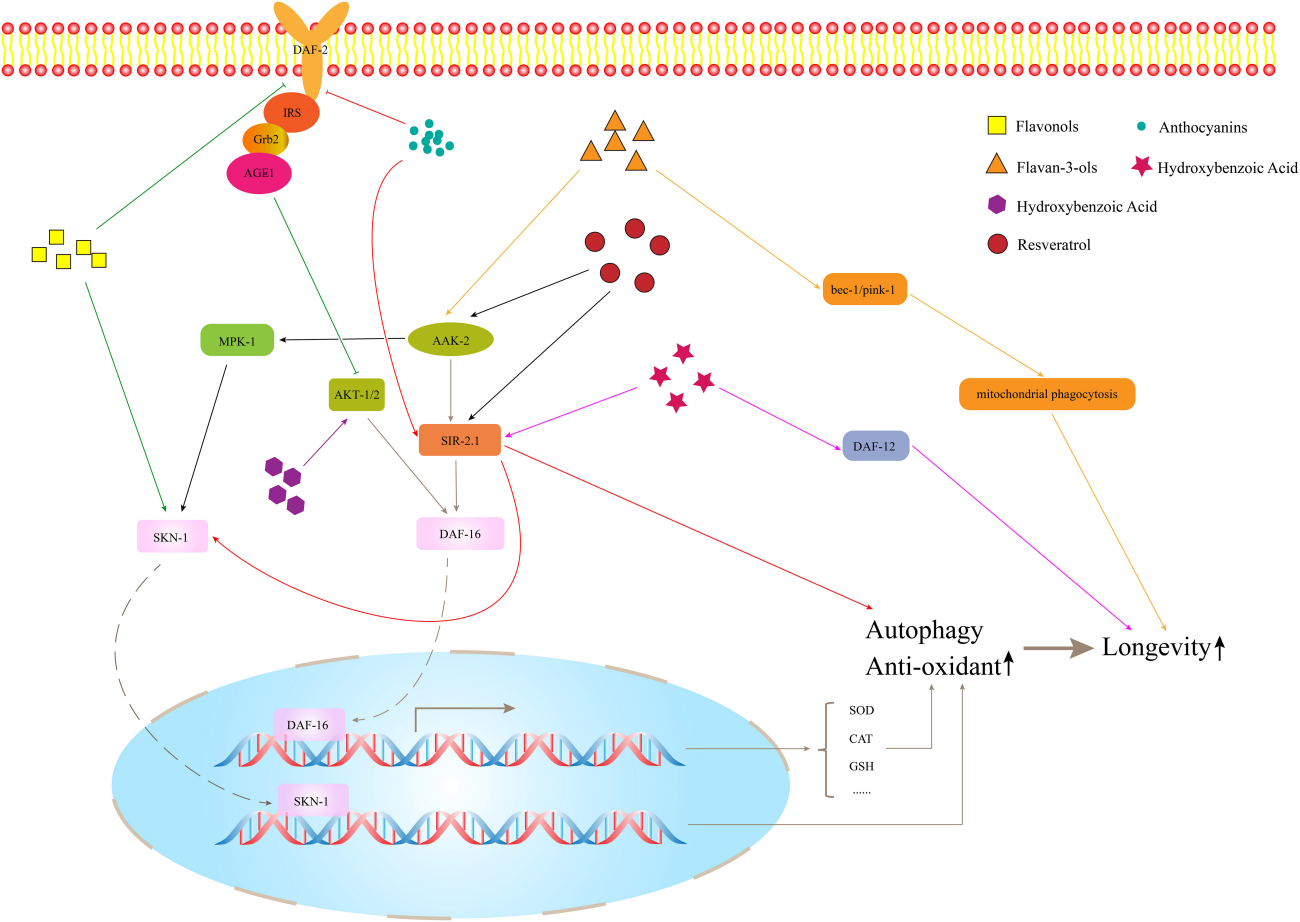
The effects of polyphenols on longevity.

Bioactive phytochemicals, such as flavonols, are abundant in fruits and vegetables, such as onions, peppers, cauliflower, and grapes. The most common flavonol is quercetin, and other common flavonols are kaempferol, myricetin, isorhamnetin, tamarixetin, morin, fisetin, apigenin, and luteolin (Adebamowo et al., 2005; Perez-Vizcaino and Duarte, 2010). Figure 2 illustrates the model of how flavonols are involved in lifespan regulation.

Quercetin, as a strong anti-oxidant, has been demonstrated to have a positive effect on longevity and stress resistance in various animal models, and its activity and mechanism have also been studied in nematodes (Pietsch et al., 2012; Proshkina et al., 2016). Several studies have confirmed that quercetin accumulates in nematodes and exhibits reactive oxygen species (ROS) scavenging activity, which might be the reason for its beneficial health effects, and this process is regulated by the transcription factor DAF-16 (Kampkotter et al., 2008; Sugawara and Sakamoto, 2020). The *C. elegans* gene, *daf-16*, is homologous to the mammalian gene for the FOXO transcription factor, which plays a key role in controlling several stress response signaling cascades, aging processes, and other important biological functions, and it is also considered as an important downstream factor of the IIS pathway, which is one of the main pathways that regulate the lifespan of nematodes. It starts from the DAF-2 insulin receptor, and it is also the ortholog of the insulin/IGF-1 receptor in *C. elegans*, through AGE-1/PI3K to AKT-1/2 and then to the downstream target DAF-16/FOXO transcription factor, to control the lifespan and metabolism of *C. elegans*. However, the conclusion is contrary. Some reports suggested that, although DAF-2 and other components of the IIS pathway mediate the anti-oxidant activity and life-prolonging effects of quercetin on nematodes, these effects seem to be independent of DAF-16 (Pietsch et al., 2009). Hence, the role of DAF-16 in quercetin-induced health effects requires further investigation. Besides DAF-16, SKN-1 and mitogen-activated protein kinase (MAPK) pathways are also involved in the process of scavenging ROS, lifespan extension, and health improvement in *C. elegans*. Quercetin also induces heat resistance by co-activating the expression and/or activity of HSF-1 and DAF-16 (Sugawara and Sakamoto, 2020). HSF orthologous, HSF-1, as a transcriptional regulator of stress-induced gene expression in worms, induces the expression of molecular chaperones. UNC-43 and SEK-1 seem to be parts of the lifespan regulation of quercetin (Pietsch et al., 2009). SEK-1 is an indispensable MAPK in innate immunity, and UNC-43 is a part of the SEK-1 upstream neuron regulation signal pathway. These two regulators belong to the MAPK pathway, a major immune signaling pathway (Troemel et al., 2006). UNC-43 is also a type II Ca^2+^/calmodulin-dependent kinase (CAMKII) that can also regulate osmotic pressure. Therefore, quercetin can be considered a multitarget nutrient.

In addition, myricetin was first discovered by Spanier for its activation of DAF-16 and its increase in the expression of its downstream gene *sod-3*, but it was found that the activation of DAF-16 was not the cause of the extended lifespan because it was found that DAF-16 activation was not correlated with the myricetin-mediated decrease in mitochondrial ROS and the increase in longevity (Grunz et al., 2012). However, further experiments proved that myricetin does exert its anti-oxidant effect through DAF-16 (Buchter et al., 2013; Sobeh et al., 2020). All these studies found that, in DAF-16 mutants, the ROS-scavenging effect of myricetin was blocked to a great extent, and the beneficial effect on lifespan also disappeared completely. This indicates that, although myricetin is a strong anti-oxidant, its effect on the lifespan of *C. elegans* is heavily dependent on DAF-16 rather than its direct anti-oxidant capacity. At present, the study of myricetin on the life extension of nematodes is limited to its regulation of the IIS pathway. In the future, additional pathways should be further analyzed in this respect, and other mechanisms for myricetin-mediating health effects should be investigated (Buchter et al., 2013).

Baicalein mainly comes from Huangqin, which is one of the commonly used traditional Chinese medicines. It has been demonstrated that baicalein mediates anti-oxidant effects by activating nuclear factor erythroid 2-related factor 2 (Nrf2) in mammalian cell lines. When it comes to *C. elegans*, SKN-1 is the homologous gene of the mammalian transcription factor Nrf2 (An and Blackwell, 2003). Similar to Nrf2, SKN-1 can also be activated by oxidative stress or exogenous bioactive substances; then, it can be transferred to the nucleus and combined with anti-oxidant response elements (AREs) of various anti-oxidant or protective gene promoter regions. This pathway can induce a variety of anti-oxidant enzymes as the key defense mechanism against oxidative stress. The lifespan of *skn-1* mutant is shortened, and the resistance to oxidative stress is reduced. SKN-1 is the direct target of the IIS pathway and MAPK pathway and has some common downstream targets with DAF-16. It is also required for dietary restriction (DR)-induced longevity because it interacts with amino acid and lipid metabolism during starvation (Dall and Faergeman, 2019). It has been reported that baicalein can modulate the lifespan and stress resistance of nematodes *via* SKN-1, but not DAF-16, a result similar to what was obtained in mammalian cell lines (Havermann et al., 2013, 2016).

## Anthocyanins

Anthocyanins are found in a wide variety of colored vegetables, fruits, and cereal, especially in various berry fruits, such as bilberries, blueberries, blackberries, blackcurrants, chokeberries, strawberries, and elderberries (Chen et al., 2013; Wallace and Giusti, 2015; Yan et al., 2017). Many studies have focused on the anti-oxidant capacity of different plant extracts rich with anthocyanins. The vast majority of plant extracts rich in anthocyanins, such as extracts of purple wheat (Chen et al., 2013), acai berry (Peixoto et al., 2016), mulberry (Yan et al., 2017), purple pitanga fruit (Tambara et al., 2018), tart cherry (Jayarathne et al., 2020), and bilberry (Gonzalez-Paramas et al., 2020), which can play their beneficial role by increasing nuclear translocation of DAF-16 and promoting the expression of anti-oxidant genes, such as *sod-3*, and the heat shock gene, *hsp-16.2*, in its downstream. Heat shock proteins (HSPs) are molecular chaperones and play important roles in the protection of molecular damage under environmental stress and have the ability to maintain proteostasis and prolong the longevity of organisms (Swindell, 2009). HSP-16.2 family is expressed under stress conditions and can be considered as stress-sensitive reporters to evaluate lifespan (Strayer et al., 2003). DAF-16 is a key protein for the anti-aging effects. Mechanistically, recent studies have found that anthocyanins could regulate the AAK-2/AMPK signaling pathway to perform its biological function (Jayarathne et al., 2020). *aak-2* is the encoding gene of AMPK in nematodes. AMPK is a regulator of cellular energy homeostasis, which is essential for the metabolic regulation of nematodes during starvation and diapause (Demoinet and Roy, 2018), it can be activated under low energy conditions and can maintain the steady state of energy, linking nutritional availability with longevity (Tullet, 2015). Overexpression of AAK-2 in nematodes prolongs its lifespan, and this effect also requires the downregulation of the IIS pathway and the upregulation and transposition of DAF-16 (Zhao et al., 2017). Additionally, mulberry anthocyanins can also activate transcription factors SKN-1/Nrf2 and PMK-1/MAPK and their downstream targets that are related to oxidative stress (Yan et al., 2017).

## Flavan-3-ols

Flavan-3-ols include catechin, gallocatechin, epicatechin, epigallocatechin, epicatechin-3-gallate, epigallocatechin-3-gallate (EGCG), theaflavin, theaflavin-3-gallate, theaflavin-3′-gallate, theaflavin-3′-digallate, and thearubigins. Flavan-3-ols are mainly found in tea, apples, wine, and cocoa (Lei et al., 2016). Figure 2 shows the effect of flavan-3-ols on the lifespan of nematode. There are many types of flavan-3-ols, but the current research has focused mostly on tea extracts and the certain classes of flavan-3-ols, such as catechinic acid (CA) and EGCG. Xiong et al. (2014) found that the black tea extracts contain a variety of flavan-3-ols, which can increase the lifespan of *C. elegans* under stress conditions, such as osmotic pressure imbalance, ultraviolet radiation, and heat stress. This effect might be mediated by the SEK-1 signaling and SIR-2.1/DAF-16/SOD-3 pathways, which could increase stress resistance. Worms simultaneously treated with catechins-rich green tea aqueous extract (GTE) and a lethal dose of pro-oxidant, i.e., juglone, showed a decreased expression of *hsp-16.2* and a significantly increased survival rate as compared with worms not receiving GTE. It suggested that GTE could enhance the anti-stress ability of nematodes and reduce oxidative damage *in vivo* (Abbas and Wink, 2014). In addition, it was found that CA, as a natural polyphenol compound, extended the lifespan and declined the age-related behaviors of *C. elegans* by regulating the mitophagy pathway related to genes of *bec-1* and *pink-1*. It was found that it can act in the role of inducing mitochondrial phagocytosis at the early stage, which was also a key period to affect the lifespan (Wu et al., 2020). Mitophagy can prevent the accumulation of dysfunctional mitochondria and prolong lifespan. EGCG is another widely studied flavane. The regulation of EGCG on the lifespan of nematodes is affected by the concentration. The effect on the organism can be described as a hormetic effect; in other words, stimulatory and inhibitory effects would be generated in low and high doses. The health effects of EGCG depend on the hormetic effect. It was found that, when the concentration was <25 μM, it could prolong the lifespan of nematodes under the stress and improve their stress ability and the partial decline of age-related physiological behavior, but it was not enough to affect the lifespan of worms under normal conditions (Brown et al., 2006; Zhang et al., 2009). When the concentration is above 800 μM, it might produce toxic effects (Xiong et al., 2018). At a suitable concentration, EGCG induced ROS in a time-resolved manner, which can temporarily increase ROS level in the early stage and activate AAK-2/AMPK, change the metabolism of NAD^+^, and then increase the expression of its downstream target protein SIR-2.1. Previous studies have found that EGCG can increase the nuclear translocation and expression of DAF-16 and activate its downstream antioxidant genes (Zhang et al., 2009; Bartholome et al., 2010). However, the upstream regulation mechanism has not been further studied. Currently, it was found that EGCG acted on SIR-2.1 instead of the IIS pathway to regulate DAF-16 (Xiong et al., 2018). Besides, EGCG can mainly restore the mitochondrial function and increase the biogenesis of early-to-mid adult worms, thus improving the redox steady state of worms. The EGCG-induced longevity of nematodes also depends on the mitochondrial function. The health effect would decrease gradually with age increases (Xiong et al., 2018). Sirtuin of *C. elegans* is the closest homolog to human SIRT1, which is encoded by the gene *sir-2.1*. It is also a conservative transcription regulator. As an NAD^+^-dependent histone deacetylase, the overexpression of sirtuin can prolong the lifespan of many species (Smith et al., 2014; Seo et al., 2015). Sirtuin can directly activate DAF-16/FOXO by deacetylation, which affects the lifespan independently of IIS (Kenyon, 2010). It can also induce autophagy by upregulating the autophagy gene and inhibiting the TOR signal together with AMPK (Ruderman et al., 2010). In addition, the anti-oxidant mechanism is activating SKN-1 and regulating lifespan through the pathway, partially overlapping with DR (Jung et al., 2017). DR is one of the most influential environmental interventions for prolonging the lifespan and healthspan of many species.

## Hydroxybenzoic Acid

Hydroxybenzoic acid is widely distributed in vegetables and fruits and can be synthesized from polyphenols by gut bacteria. It has been confirmed to activate Nrf2 (Juurlink et al., 2014), suggesting that it might have the anti-aging effects on nematodes *via* the Nrf2 signaling pathway. Furthermore, 4-hydroxybenzoic acid could extend the lifespan of nematodes through the activation of DAF-16/FOXO mediated by SIR-2.1/SIR-2, which demonstrated no relationship with DR and IIS pathway. It can also increase the stress resistance under osmotic, heat, and oxidative stress conditions (Kim et al., 2014). By bioinformatics analysis, aspirin was found to alter the expression of genes, which are involved in fat metabolisms, such as *acs-2, ech-1.2*, and *cpt-5*, which can extend the longevity of *C. elegans* through the activation of DAF-12 and DAF-16 (Huang et al., 2017). DAF-12 is a nuclear hormone receptor that can be initiated by insulin/IGF-1 and TGF-β and plays an important role in metabolism, longevity, and reproductive development in *C. elegans*. As is known to all, the primary component of aspirin is salicylic acid. As an isomer of hydroxybenzoic acid, it suggests that hydroxybenzoic acid might influence the expression of genes involved in anti-oxidation and fat metabolism.

## Hydroxycinnamic Acid

Hydroxycinnamic acid and its derivative, caffeic acid, are abundant in tea leaves, red wine, and coffee. It was reported that extracts of green coffee beans (GCEs), which are composed mainly of chlorogenic acid (CGA) and its derivative, 5-caffeoylquinic acid (5-CQA), have beneficial effects on longevity and reproduction in *C. elegans*. The study also indicated that, compared with CGEs rich in pure 5-CQA, the CGEs rich in 5-CQA have a stronger anti-aging effect, which strongly supports that it might be a better choice to use the mixture of bioactive compounds instead of just one single bioactive molecule (Amigoni et al., 2017). At the same time, it was also found that CGA and its isomers, such as 5-CQA and 4-caffeoylquinic acid (4-CQA), acted on the upstream of AKT in the IIS pathway and then exerted their life-prolonging and anti-aging effects mainly *via* DAF-16 and its downstream stress factors, HSF-1, SKN-1, and HIF-1 (Zheng et al., 2017). Moreover, *p*-coumaric acid, another derivative of hydroxycinnamic acid, can enhance the ability to resist SKN-1-mediated oxidative stress and OSR-1-mediated osmotic stress (OSR-1 can negatively regulate the activity of the MAPK pathway) (Yue et al., 2019).

## Lignans

Six lignans were isolated from *Arctium lappa* seeds, and all of them were found to have anti-aging properties and to upregulate the expression of *daf-16* and *jnk-1* (Su and Wink, 2015). *jnk-1* is considered as a positive regulator of *daf-16*, which indicates that lignans have the life-promoting activity through the JNK-1/DAF-16 cascade. Sesamin is a major lignan constituent of sesame and possesses various health-promoting effects. That sesamin could not only prolong the life of nematodes but could also reduce the toxicity of Alzheimer's disease (AD) β-amyloid (Aβ) plaque (Keowkase et al., 2018). It was also found that the resistance of nematodes to physical stress and some pathogenic bacteria could not be enhanced by sesamin, but it could protect nematodes from oxidative stress caused by toxins, which is partly due to the indirect hormetic effect of sesamin. Besides, it was found that sesamin could play the anti-aging role *via* the genes constituting the IIS pathway (*daf-2* and *daf-16*) and MAPK pathway (*pmk-1* and *skn-1*) (Yaguchi et al., 2014). PMK-1 is a kinase that plays an important role in immune defense and longevity in the MAPK pathway. In addition, sesamin can also act as a mimic of DR. Sesamin depends on SIR-2.1/SIRT1, AAK-2/AMPK, an autophagic modulator BEC-1, and *daf-15*, which encodes the target of TOR-binding partner raptor, to promote longevity (Yaguchi et al., 2014; Nakatani et al., 2018). The inhibition of the TOR pathway is another well-known intervention method to prolong lifespan. DR might induce autophagy and activate DAF-16 by inhibiting TOR kinase (Cypser et al., 2013). BEC-1 is necessary for the longevity induced by overexpression of *sir-2.1*. SIRT1, TOR, and AMPK are currently known as signaling pathways associated with DR. Unlike other DR analogs, sesamin is likely to be involved in almost all of the known DR-related pathways, which can prolong lifespan.

Another lignan, i.e., pinoresinol has been observed to increase the nuclear translocation of DAF-16, but it has no effect on the longevity of nematodes and has no regulating ability to the stress resistance and oxidation resistance. Although it shows strong oxidation resistance *in vitro*, its functional effects in organisms need further study at a molecular level (Koch et al., 2015).

## Stilbenes

The most important representative of stilbene compounds is resveratrol, which is mainly derived from grape skins, grape seeds, red wine (Salehi et al., 2018), blueberries, peanuts, and some traditional Chinese herbal medicines, such as rhubarb (Malaguarnera, 2019), and polygonum cuspidatum (Zhang, 2006). Resveratrol is usually recommended as a dietary supplement to maintain redox balance and to delay aging (Desjardins et al., 2017).

The activation of sirtuins is considered to be an important mechanism of resveratrol-mediated longevity. Research found that resveratrol can activate SIR-2.1 and then prolong the life of nematodes by regulating *bec-1* to induce autophagy (Morselli et al., 2010). Lee et al. (2016) found that resveratrol does not need to exert its health effects through DAF-16 after activating SIR-2.1, which indicates that there might be other regulatory pathways downstream of SIR-2.1, whereas Yoon et al. (2019) found that SIR-2.1 relies on DAF-16 for its function, so the role of DAF-16 in resveratrol-induced longevity needs further study. As research progresses, scientists have learned more about the mechanism of how resveratrol works to delay aging. The effect of resveratrol on life extension might not work entirely in a sirtuin-dependent way. As a DR analog, resveratrol can prolong lifespan through AAK-2, a key factor in the AMPK pathway, and without the association of DAF-16. Similar to SIR-2.1, MPK-1 is also one of the key regulators for lifespan extension (Yoon et al., 2019). However, its contribution to resveratrol-mediated life extension is completely independent of SIR-2.1, and they have different downstream regulatory genes. MPK-1 is also known as human ERK homo that acts by promoting downstream SKN-1 nuclear translocation and is first identified as a longevity factor (Okuyama et al., 2010). Resveratrol can alleviate the damage caused by ROS and prolong the life of nematodes under pressure (Chen et al., 2013). In addition, the two newly synthesized resveratrol derivatives have stronger biological and anti-oxidant activity than resveratrol. Their strong anti-oxidant ability can also regulate DAF-16, SKN-1, SIR-2.1 in the redox activity signal pathway (Fischer et al., 2017).

## Tannins

Tannic acid (TA) belongs to the hydrolyzable tannins, containing five digallic acid residues covalently linked to a central glucose molecule, and it can precipitate protein. As a strong anti-oxidant, the observed increase in heat stress resistance and oxidative stress resistance is not due to its ability to directly remove oxygen free radicals but its ability to act as a stimulus to activate the anti-oxidant system of the body (Saul et al., 2010, 2011). Studies have shown that a low concentration of TA might simulate mild pathogenic stress, strengthen the SEK-1-based pathogen defense system, inhibit the potentially harmful effects of TA, and induce nematodes to prolong their life effectively (Saul et al., 2010). In addition, TA itself does not reduce the food intake of nematodes, but exerts the molecular regulation through *eat-2* in the DR pathway or precipitates and combines nutritional proteins and digestive enzymes (*eat-2* mutant suffered from insufficient food intake due to decreased pharyngeal pumping). It is worth noting that the concentration range of health effects of TA is relatively narrow, so it is also very important to find a suitable concentration to treat *C. elegans*. Unlike TA, ellagic acid (EA) can be used as a chemical repellent to reduce the feeding behavior of nematodes and prolong the lifespan of nematodes by its strong antibacterial ability (Saul et al., 2011). Oenothein B (Chen et al., 2020) and pentagalloyl glucose (Chen et al., 2014) extracted from eucalyptus leaves can prolong healthy life by regulating multiple targets. They can regulate the IIS pathway *via age-1* and *daf-16*, the DR pathway *via eat-2* and *sir-2.1*, and mitochondrial electron transfer chain *via isp-1* to promote healthy life, including reducing age pigment and ROS accumulation and improving exercise flexibility, heat stress tolerance, and lifespan. *isp-1* is one of the genes encoding mitochondrial electron transport chain components, and the deletion of *isp-1* exists in the respiratory chain complex III. Their mechanism of action might be the same because of their similar structure.

## Conclusions

Before studying the signaling pathway implicated in a certain disease in a model organism, some researchers first test this pathway in suitable cell lines. For example, resveratrol was found in the generation of different effects, such as promoting proliferation in mesenchymal stem cells, with possible involvement of the ERK/GSK-3β pathway (Yoon et al., 2015). Nematodes were further utilized to test the MPK-1 (an ERK homolog) signaling (Yoon et al., 2019). When we treat nematodes with plant extracts, we can first analyze each extract component using mass spectrometry, high-performance liquid chromatography, or similar methods; this analysis might help to identify the key components responsible for the biological effects.

At present, researchers rarely treat *C. elegans* with polyphenols, such as flavanones and isoflavones; therefore, it is unknown whether or not these polyphenols have direct effects on *C. elegans*. However, many studies demonstrated that the abovementioned polyphenols can act on homologous genes of *C. elegans* in other species and that these genes are involved in aging and regulation of lifespan. Flavanones, of which the representative molecule is hesperidin, are mainly found in citrus plants and have been demonstrated to be able to reduce oxidative stress caused by a high-fat diet in mice and to slow down the aging process in old-aged rats. Some studies found that one of the targets of flavanones in animals is Nrf2, whereas *C. elegans* has an Nrf2-homolog gene, SKN-1 (Ferreira et al., 2016; Barreca et al., 2017; Habtemariam, 2019; Miler et al., 2020). Isoflavones, such as genistein and daidzein, are generally regarded as phytoestrogens; there is evidence that Nrf2 is also one of the downstream targets of isoflavones and that it can also regulate fat metabolism in rats with diet-induced obesity, acting through the AMPK pathway (Li and Zhang, 2017; Krizova et al., 2019). In conclusion, further studies should be conducted to test whether these polyphenols have beneficial effects on *C. elegans*.

## Prospects

To the best of our knowledge, in the study of total polyphenols in plants, especially medicinal plants, their functions, the bioactive substances, and molecular mechanisms for prolonging lifespan and delaying senescence have not received enough attention. For example, mulberry leaves have been widely used in traditional Chinese medicine and folk dietary therapy for their outstanding effects of detoxifying the liver, improving eyesight, and prolonging life. It is believed that mulberry leaf extracts used in traditional Chinese medicine have anti-oxidant and hepatoprotective effects, and those two activities are related to mitochondria function (Meng et al., 2020). Liver tissue contains a large number of mitochondria, and the fatty acids are activated into ester-acyl-coenzyme A, which is metabolized by β oxidation in mitochondria. Acetyl-coenzyme A and fat synthetases required for fatty acid synthesis come from mitochondria. At present, mulberry leaf extract has been confirmed to have beneficial effects on several diseases, such as cancer, type 2 diabetes, and obesity. In addition, modern medicine experiments have proved that mulberry leaf extract can delay aging in mice (Lim et al., 2013; Turgut et al., 2016). Mulberry leaf extract is an effective and natural free-radical scavenger and anti-oxidant, but the research on mulberry leaf extract and mulberry leaf polyphenol is limited to its anti-oxidant activity *in vitro*, and its specific mechanisms of action have not been elaborated. Moreover, the activities of mulberry leaf polyphenols have not yet been associated with any specific physiological functions. Besides, nematodes being treated by the combination of two extracts from different plants revealed stronger effects than the treatment with only either of the single extract. A recent study shows that nematodes treated with the mixtures of blueberry and apple peel extracts have a longer lifespan than those treated with only one substance (Song et al., 2020a,b). Could mulberry leaf extract exert the effects observed in traditional Chinese medicine by regulating fat metabolism? What are the specific anti-aging mechanisms of mulberry leaves in *C. elegans*? Could the combinations of mulberry leaves, polyphenols, and other polyphenols, or other bioactive substances play their beneficial roles more significantly, and what are their mechanisms? We would like to answer these questions by conducting further research.

## Author Contributions

ZQ and NL conceived the idea and wrote the manuscript with input from LL, PG, and SZ. PG and PW prepared the figures. LL, PW, and SZ prepared the tables. All authors edited and approved the final manuscript.

## Conflict of Interest

The authors declare that the research was conducted in the absence of any commercial or financial relationships that could be construed as a potential conflict of interest.
